# Pharmacist Services

**DOI:** 10.3390/pharmacy7040141

**Published:** 2019-10-10

**Authors:** Jon C. Schommer, Anthony W. Olson

**Affiliations:** 1 College of Pharmacy, University of Minnesota-Twin Cities, Minneapolis, MN 55455, USA; 2 College of Pharmacy, University of Minnesota-Duluth, Duluth, MN 55812, USA

Welcome to the “Pharmacist Services” special issue in the journal Pharmacy, an open access journal with a focus on pharmacy education and practice. In 2018, an invitation was dispersed to scholars in the pharmacist services domain asking them to submit a manuscript to this special issue no later than 31 July 2019. We invited these colleagues to think about a full breadth of topics including, but not limited to: (1) The history and development of pharmacist services, (2) service settings, (3) service management, (4) service profitability, (5) service recovery, (6) service relationships, (7) service quality, (8) service tailoring, (9), service design and standards, (10) service performance, and (11) service evaluation. We sought manuscripts of all types including: (1) reviews, (2) commentaries, (3) idea papers, (4) case studies, (5) demonstration studies, and (6) research studies. The call for papers was delineated using ideas published by renowned experts in the services management and marketing domains including: Teresa Swartz, Dawn Iacobucci, Roland Rust, Richard Oliver, Valerie Zeithaml, and Mary Jo Bitner. With this foundational context described and the invitations sent, we waited to learn about what would be submitted in a timeframe of just several months.

We are pleased to report that over 30 articles have been published in this special issue and represent the work of about 100 scholars in this domain. To receive such a response from busy colleagues in such a short time-frame is incredible. The overall goal of this special issue on “Pharmacist Services” is to give the reader a state-of-the-art synopsis of the pharmacist services domain in the year 2019. To accomplish this goal, we sought papers that address the social, psychosocial, political, legal, historic, clinical, and economic factors that are associated with pharmacist services. Papers that translate concepts from other domains into the pharmacist services realm were welcomed. As we review the articles in this special issue, a great deal can be learned about (1) pharmacist professionalism, (2) pharmacist practice, and (3) pharmacist progression.

**Pharmacist Professionalism**

The articles reveal that pharmacist services vary by country, design, delivery environment, payment schemes, end-user, requisite training, regulatory standards, and more. As different as pharmacist services are, they are all linked by the individuals who provide them. Whether dispensing or immunizing, reconciling medication lists or performing medication management, pharmacists use their expertise related to medications to pursue excellent, affordable, and accessible healthcare for a common beneficiary: their patients. Pharmacist professionalism is a driving force for translating pharmacists’ expertise into medication use, for helping people achieve medication experiences that are life enhancing.

**Pharmacist Practice**

Another emergent theme for pharmacist services described in this special issue is to view them through the Joint Commission of Pharmacy Practitioners (JCPP) Pharmacist Patient Care Process (PPCP), which was created and is supported by pharmacist organizations representing managed care, education, consultants, in-patient practice, outpatient practice, governmental regulatory bodies, and more. This model identifies a consistent process of care in the delivery of patient care services consisting of the following five steps: (1) Collect, (2) Assess, (3) Plan, (4) Implement, and (5) Follow-up: Monitor and Evaluate. The articles in this special issue give readers a state-of-the-art snapshot regarding the diversity of pharmacist services through the prism of the JCPP Pharmacist Patient Care Process.

**Pharmacist Progression**

Finally, many articles in this special issue reveal how pharmacist services can progress in (1) societal relevance, (2) innovative delivery, (3) integration into broader systems, (4) enhanced image to both payers and consumers, and (5) growth into new roles and markets. The articles reveal the overwhelming opportunities that pharmacists can embrace and fill such as: (1) Medication optimization, (2) wellness and prevention, (3) chronic care management, (4) acute care management, (5) patient education, (6) care transitions, (7) population health, (8) emergency preparedness, (9) health informatics, and (10) patient-centered, living-in-place care.

We trust that you will find the articles in this special issue not only informative, but inspiring as well. We greatly appreciate the colleagues who worked to meet a short deadline and were willing to share their work with us. We also wish to thank the editorial staff who coordinated the review and publishing processes. Their professionalism is highly valued. Finally, we wish to thank you, the reader. Please apply the ideas in these articles to your work. Expand upon them. Challenge them. And then, share your work with us.

